# Granulocyte-Monocyte Apheresis in Steroid-Dependent, Azathioprine-Intolerant/Resistant Moderate Ulcerative Colitis: A Prospective Multicenter Study

**DOI:** 10.1155/2017/9728324

**Published:** 2017-12-21

**Authors:** Gianni Imperiali, Arnaldo Amato, Maria Maddalena Terpin, Ivo Beverina, Aurora Bortoli, Massimo Devani, Chiara Viganò, Study Group on IBD (GSMII)

**Affiliations:** ^1^Gastroenterology Division, Valduce Hospital, Como, Italy; ^2^Gastroenterology Division, Legnano Hospital, ASST Ovest Milanese, Legnano, Italy; ^3^Blood Transfusion Center, ASST Ovest Milanese, Legnano Hospital, Legnano, Italy; ^4^Gastroenterology Division, Rho Hospital, ASST Rhodense, Rho, Italy; ^5^Gastroenterology Division, San Gerardo Hospital, ASST Monza, Monza, Italy

## Abstract

**Background:**

Granulocyte-monocyte apheresis has been proposed for the treatment of ulcerative colitis, although it is limited by costs and variability of results.

**Aim:**

To assess effectiveness of granulocyte-monocyte apheresis in patients with steroid-dependent, azathioprine-intolerant/resistant moderate ulcerative colitis.

**Methods:**

Consecutive patients fulfilling inclusion criteria were prospectively enrolled, treated by apheresis, and followed up for 12 months. The primary end point of the study was steroid-free clinical remission at 12 months, with no need for biologic therapy or surgery.

**Results:**

From January to December 2013, 33 patients were enrolled. After one year of follow-up, 12 (36%) patients had clinical remission, were steroid-free, and had no need for biological therapy or surgery; 3 (9%) cases showed a clinical response (but not clinical remission). Moreover, 12 (36%) patients required biologic therapy, 4 (12%) underwent colectomy, and in the other 2 (6%) a reduction, but not withdrawal, of steroid dose was achieved.

**Conclusions:**

Our study shows that a standard course of granulocyte-monocyte apheresis is associated with a 36% steroid-free clinical remission in patients with steroid-dependent, azathioprine-intolerant or resistant moderate ulcerative colitis. Apheresis might represent an alternative to biologic therapy or surgery in this specific subgroup of patients. This trial is registered with Clinicaltrial.gov NCT03189888.

## 1. Introduction

The choice of treatment for patients with ulcerative colitis (UC) is related to the extent, clinical, and endoscopic severity of the disease, along with the number and severity of relapses. Patients with no response to conventional therapies (e.g., mesalamine, steroids, and immunosuppressive agents) are typically indicated to long-term biological treatments or surgery. Both these options are encumbered by high costs and significant incidence of side effects. Patients with UC likely have elevated and activated granulocytes, and, in case of active disease, infiltration of a large number of granulocytes and macrophages into the bowel mucosa can be observed. Infiltrating leukocytes can release degradative enzymes, oxygen derivatives, and proinflammatory cytokines, causing bowel injury and promoting further inflammation. Based on the hypothesis that the reduction of this excess of activated granulocytes and monocytes/macrophages may be beneficial, apheresis has been proposed as a strategy to promote remission in active UC. This treatment presents some strengths, mainly as a safety concern, but also some limits, that is, elevated costs and unclear efficacy.

In detail, the first studies published in Japan showed remission or response rates up to 60–80% [1–3]; conversely, in a subsequent study enrolling a large number of patients to compare GMA to placebo, no significant difference was observed in terms of clinical response [4]. Among the factors to explain this substantial difference, a role may be played by the heterogeneity of patients included into the studies, mainly regarding the severity and extent of disease. Up to now, the subgroups of patients in which the GMA could have represented the most cost-effective strategy have not been identified yet.

The aim of the present study was to evaluate the efficacy of GMA in a homogeneous subgroup of patients with moderate steroid-dependent, azathioprine-intolerant or resistant UC, as a potential alternative to long-term biologic therapy or surgery.

## 2. Methods

Consecutive patients with steroid-dependent, azathioprine-intolerant or resistant moderate UC were enrolled in this prospective multicenter study, performed in six Gastroenterology Departments of community hospitals in Northern Italy, with the approval of the Ethics Committee of the coordinating center, and informed consent was obtained for each patient. All patients with a diagnosis of UC, defined at least one year before confirmed by clinical and endoscopic-histological data, were considered for study enrollment. Within this population, patients with moderate UC, defined by a colitis activity index or Rachmilewitz index (CAI) ranging from 6 to 11, with steroid dependence and azathioprine intolerance or resistance were identified. In detail, steroid dependence was defined as the inability to reduce the steroid dose below 10 mg of prednisone or equivalent per day within 3 months after therapy initiation or exacerbation of disease within 3 months after the suspension of the steroid cycle. Azathioprine intolerance and resistance were defined as treatment interruption due to drug-related adverse event and no response after at least 4 months of therapy at conventional doses (2.0–2.5 mg/kg), respectively.

Patients with rectal involvement only, prior colonic surgery, and previous biologic treatment were excluded from the study.

GMA was performed using the Adacolumn® leukapheresis system (Otsuka Pharmaceuticals, Tokyo, Japan).

Demographic data, smoking status, medical history, date of diagnosis, extension of disease, and pharmacological history were recorded. All patients had undergone colonoscopy in two months before enrollment, with Baron index calculated; their stool cultures had to be negative in the month preceding GMA, and for each patient, complete blood count, C-reactive protein, and CAI were recorded at enrollment.

Enrolled patients underwent five weekly sessions of GMA; biochemical and clinical parameters were recorded at each session. All patients were reassessed at 3, 6, and 12 months with biochemical (at months 6 and 12) and clinical parameters. An endoscopic control was scheduled at twelve months from the enrollment.

During the study period, patients could continue ongoing mesalamine therapy, if in treatment, without any change in the dosage. Steroid tapering was started between the second and fifth apheresis session, with a 5 mg decrease per week.

Clinical remission was defined as CAI < 4, with steroid-free remission and no need for biologic therapy or surgery at the end of the 12-month follow-up. Clinical response was considered as a CAI decrease of at least 3 points and the reduction of steroid dosage.

### 2.1. Statistics

Categorical variables were summarized using frequencies and percentages, while quantitative variables were summarized using means and standard deviations (SD). Chi-squared test was used to compare categorical variables, whereas Student's *t*-test was used for continuous variables. A *p* value < 0.05 was considered statistically significant.

## 3. Results

From January to December 2013, 33 consecutive patients (18 males, mean age 44.3 years, range 27–78) were enrolled in the study.

Overall, 20 and 13 patients had pancolitis and left-sided colitis, respectively. The mean duration of disease was 7 years (range 2–21). The mean CAI at enrollment was 8.1 (range 6–11), and the mean Baron index was 2.1 (range 1–3); three patients (9%) were smokers. No patient suspended the GMA sessions for adverse events, whereas one complained of headache during the procedure (treated with acetaminophen).

After 12 months of follow-up, steroid-free clinical remission (CAI < 4), with no need for biologic therapy or surgery, was obtained in 12 patients (36%), whereas the clinical response was observed in other 5 patients (15%), 2 of which (6%) reduced but did not suspend the steroid and three (9%) obtained a CAI decrease of three points, but without clinical remission.

Moreover, 12 patients (36%) had to start biologic treatments (8 in the first 3 months, 2 between the fourth and sixth month, and 2 after the sixth month after GMA initiation). Four patients (12%) underwent surgery (all within 5 months from the enrollment).

All 12 patients with clinical remission at one year had already achieved it at 3 months, and all improved their Baron index at follow-up colonoscopy, with a posttreatment score of zero or one.

Responders to GMA were significantly younger than nonresponders (mean age 39.5, SD 12.4 years versus 50.8, SD 14.0, *p* = 0.027). No significant difference between responders and nonresponders to GMA was observed as concerns gender, smoking status, disease extension and duration, baseline CAI, and Baron index (Table 1).

## 4. Discussion

The present study shows that a brief cycle of GMA in patients with steroid-dependent, azathioprine-intolerant or resistant moderate ulcerative colitis is associated with a 36% steroid-free clinical remission and a 15% clinical response after 12 months of follow-up.

To our knowledge, this is the first report in the literature in the efficacy of GMA in this selected subgroup of UC patients. The first Japanese studies on GMA reported proportions of response or clinical remission ranging from 60 to 80% [1–3] in UC patients overall. In 2008, Sands and colleagues [4] published a study including more than 200 patients, in which a clinical response was achieved in 44% of those treated by active column and in 39% of those treated by placebo, with no significant difference between the two treatments. Although considerable for the large number of patients included, the above study is characterised by some biases, mainly due to the high number of patients lost at follow up and to questionable inclusion criteria, mainly regarding the severity and extent of disease.

A subsequent meta-analysis [5], which considered six randomized trials evaluating GMA versus steroid (only one fully blinded), for a total of 549 patients, demonstrated a clinical benefit of GMA over traditional therapies and confirmed the low incidence of adverse events. However, these data had some criticisms and were not totally bias-free [6].

Along with an overall inconsistency of results on GMA effectiveness, another open issue is related to different results observed in Japanese series [7] as compared to Western ones, whose explanation could be represented by different ethnic features or disease course in the two population [8].

Considering the poor evidence available, it may be reasonable to identify selected subgroups of UC patients in which the cost-effectiveness of GMA could be tested. This issue is relevant, because in previous studies, responders to GMA showed a better long-term clinical outcome by avoiding corticosteroids during their first active UC phase [9]. Some studies [9, 10] evaluated recently diagnosed UC patients with mild clinical activity, where GMA was associated with a good clinical response, but a not so profitable cost-benefit ratio. Consistent results have been drawn for GMA in the maintenance of remission [11]. Furthermore, other fields of application have been represented by subgroups where the safety profile is very important, such as adolescents [12], pregnant women [13], and patients with cytomegalovirus superinfection [14].

Saniabadi et al. [15] recently identified best responder patients at first episode, who are more often drug naive and have a short duration of IBD [16], followed by steroid naive patients [12, 17–19], with an efficacy rate of almost 100% and over 85%, in the first episode cases [16] and in steroid naive cases [17, 18], respectively. Moreover, Yokoyama et al. [20] suggested that GMA should be proposed soon after a clinical relapse, especially in subjects with a short duration of active disease. Furthermore, other authors identified patients with more severe endoscopic lesions [16, 19, 21, 22] and long duration of active disease and assumption of more than one conventional drugs as worst candidates for GMA [16, 21–23].

To postulate the hypothesis of the present study, we considered the results coming from Spain [24], where clinical remission was observed in 37% of patients steroid-dependent or resistant [24], and from Italy, where these rates were even higher [25]. These data stand for a high cost-effectiveness of GMA in such patients in comparison with the continuation of steroid therapy [26, 27]. We considered a homogeneous cohort of nonresponders to conventional therapies (steroid dependent and azathioprine intolerant or resistant). These patients would be alternatively candidates to long-term biologic therapy or surgery, which are both associated with high costs and side effects.

In our series, the only predictive factor for response to GMA was younger age, whereas no difference in treatment response was disclosed for gender, extent and duration of the disease, previous therapy, smoking status, and clinical and endoscopic activity.

Moreover, we observed an excellent safety profile, with only one patient experiencing episodic headache during a GMA session, which was effectively treated by acetaminophen; he did not need to discontinue the treatment course. These data are consistent with the data from literature, which report GMA-related adverse events in less than 10% of the cases; this finding is particularly striking, when compared to a frequency of side-effects approaching 45% for conventional therapies [1]. Moreover, GMA-related adverse events are mild, mainly represented by venous cannulation and headache, which do not require treatment discontinuation.

The main limitation of the study is represented by the small sample of patients enrolled, but, probably due to the conflicting data on the efficacy of GMA, this treatment is not very common in our country. Moreover, inclusion criteria were very strict and reduced the number of eligible subjects. On the other hand, the strengths are the homogeneity of the patients enrolled, the objectivity of the outcome selected (CAI < 4, need of biologic therapy or surgery), and the separation of patients with clinical remission and response, in order to provide detailed information on treatment efficacy.

In conclusion, in our opinion, a short course of GMA might be proposed as a safe and effective treatment option for steroid-dependent, azathioprine intolerant or resistant UC patients, before referring them to long-term biologic therapy or surgery, which are characterised by significantly higher costs and side effects.

## Figures and Tables

**Table 1:** Patients' features.

	Responders (*n* = 12)	Nonresponders (*n* = 21)	*p*
Age, median (SD)	39.5 (12.4)	50.8 (14.0)	0.027
Sex			
Male	7	11	0.974
Extension of disease			
Pancolitis	7	15	0.639
Left-sided	5	6	
Extension of disease	7.25 (5.56)	9.33 (4.54)	0.252
CAI, median (SD)	8.00 (2.78)	8.29 (2.26)	0.764
EAI, median (SD)	2.00 (0.74)	2.01 (0.62)	0.696

CAI: colitis activity index; EAI: endoscopic activity index.
